# The role of hepatic transferrin receptor 2 in the regulation of iron homeostasis in the body

**DOI:** 10.3389/fphar.2014.00034

**Published:** 2014-03-06

**Authors:** Christal A. Worthen, Caroline A. Enns

**Affiliations:** Department of Cell and Developmental Biology, Oregon Health and Science UniversityPortland, OR, USA

**Keywords:** transferrin receptor 2, hereditary hemochromatosis, iron homeostasis, hepcidin, liver

## Abstract

Fine-tuning of body iron is required to prevent diseases such as iron-overload and anemia. The putative iron sensor, transferrin receptor 2 (TfR2), is expressed in the liver and mutations in this protein result in the iron-overload disease Type III hereditary hemochromatosis (HH). With the loss of functional TfR2, the liver produces about 2-fold less of the peptide hormone hepcidin, which is responsible for negatively regulating iron uptake from the diet. This reduction in hepcidin expression leads to the slow accumulation of iron in the liver, heart, joints, and pancreas and subsequent cirrhosis, heart disease, arthritis, and diabetes. TfR2 can bind iron-loaded transferrin (Tf) in the bloodstream, and hepatocytes treated with Tf respond with a 2-fold increase in hepcidin expression through stimulation of the bone morphogenetic protein (BMP)-signaling pathway. Loss of functional TfR2 or its binding partner, the original HH protein, results in a loss of this transferrin-sensitivity. While much is known about the trafficking and regulation of TfR2, the mechanism of its transferrin-sensitivity through the BMP-signaling pathway is still not known.

## INTRODUCTION

Iron is a necessary element for organisms, playing a role in vital processes such as the electron transport chain, the distribution of oxygen throughout the body by hemoglobin, and as a cofactor in numerous enzymatic reactions. Despite its importance, excess iron can be very toxic to the cell. Its participation in the Fenton reaction results in the formation of free radicals, which can wreak havoc by oxidizing lipids, cleaving proteins, and damaging DNA and RNA. Because of this duality, cells and organisms have evolved exquisite control mechanisms to ensure that the proper amount of iron is present, that excess iron is stored in non-toxic forms, and that within the body iron is chaperoned both outside and within cells.

The body needs 20–30 mg of iron per day for erythropoiesis, the vast majority of this iron is acquired through the efficient recycling of red blood cells by macrophages (Cook et al., 1973). The remainder is met by the dietary absorption of ~2 mg of iron per day (Cook et al., 1973). The iron importer, divalent metal transporter 1(DMT1) is located on the apical side of enterocytes in the small intestine and transports dietary iron, which has been reduced to ferrous iron by the ferrireductase, Dcytb, into the cell (Gunshin et al., 1997; Andrews, 1999; McKie et al., 2001). Once in the cell, iron that is not exported can be stored in the iron storage protein, ferritin, which is a 24-subunit protein with a hollow core that can oxidize and store up to 4500 atoms of ferric iron (Koorts and Viljoen, 2007). Enterocytes are quickly turned over by sloughing into the lumen of the intestine. Thus, enterocyte iron stored in ferritin is lost if not mobilized beforehand. Iron transport into the bloodstream is accomplished through the basolateral iron exporter, ferroportin (FPN; Abboud and Haile, 2000; Donovan et al., 2000; McKie et al., 2000), which facilitates the transport of ferrous iron and its subsequent oxidation by hephaestin and loading into transferrin (Tf) the iron transport protein in plasma (Vulpe et al., 1999; Nemeth et al., 2004). This process is at the crux of body iron regulation; amount of FPN on the basolateral membrane determines whether iron is transported into the body and FPN levels are regulated by the peptide hormone, hepcidin.

Hepcidin is regulated in and secreted by hepatocytes in the liver. This 25 amino acid peptide is the result of two cleavages of the 87 amino acid precursor, a prepropeptide cleavage of the signal sequence, and a propeptide cleavage by furin (Valore and Ganz, 2008). Serum and urine levels correspond to mRNA levels, indicating that in many circumstances hepcidin is regulated at the level of transcription (Kemna et al., 2008). Serum hepcidin binds FPN and stimulates the internalization and subsequent degradation of FPN, thus making it a negative regulator of total body iron influx (Nemeth et al., 2004).

In addition to its role as a master regulator of dietary iron absorption, hepcidin plays a role in immunity. Macrophages can also secrete hepcidin in response to inflammation through the TLR-4 signaling pathway (Liu et al., 2005; Peyssonnaux et al., 2006). Increased hepcidin prevents both absorption of iron from the gut, and the release of iron from iron-recycling macrophages, restricting the use of iron by invading pathogens. High levels of hepcidin in diseases of chronic inflammation cause the anemia of chronic disease (ACD). In contrast, abnormally low levels of hepcidin expression leads to iron-overload. Genetic mutations that reduce hepcidin expression result in the disease, HH.

Of the four types of HH, three are the result of low levels of hepcidin expression; Type I, (HFE mutation), Type II a and b [hemojuvelin (HJV) and hepcidin mutations respectively], and Type III (TfR2 mutation). Type IV is a result of mutations in FPN itself, making it insensitive to hepcidin regulation. Type I and III mutations cause a slow accumulation of iron over the individual’s lifetime and a disease phenotype starting in adulthood, while Type II a and b mutations have the more severe phenotype of Juvenile Hemochromatosis. Left untreated, HH leads to iron accumulation in the liver, heart, pancreas, and joints leading to cirrhosis, arrhythmias, diabetes, and arthritis (Weber, 1931; Schumacher, 1964; Cecchetti et al., 1991). The slow onset of adult HH clearly shows that fine-tuning of the daily influx of iron is necessary for normal iron homeostasis.

TfR2 is the putative “iron sensor” that fine-tunes this system, based on its ability to bind and be stabilized by iron-bound Tf (Johnson and Enns, 2004; Robb and Wessling-Resnick, 2004; Johnson et al., 2007). In addition, its mutation leads to low hepcidin expression (Kawabata et al., 2005; Nemeth et al., 2005), as well as an inability to respond to acute iron-loading (Girelli et al., 2011).

## BINDING PARTNERS, REGULATION, AND TRAFFICKING OF TfR2

The structure of TfR2 and its known binding partners provide clues to its function. The interesting features of TfR2 start in its structural similarity to the originally characterized transferrin receptor 1 (TfR1). They are both type II membrane proteins that function as a disulfide-linked dimer and share 66% homology in their ectodomain (Kawabata et al., 1999). Both TfR1 and TfR2 bind Tf and many of the amino acids involved in the binding of TfR1 to Tf are conserved in TfR2 (Giannetti et al., 2003). TfR1 binds iron-loaded Tf (holo-Tf) with a K_D_ of 1.1 nM and is responsible for the endocytosis of holo-Tf into acidic compartments. This iron is then released from Tf, reduced, and transported across the endosomal membrane by the metal transporters DMT1 and Zip14 (Dautry-Varsat et al., 1983; Rao et al., 1983; West et al., 2000; Zhao et al., 2010). While TfR2 is capable of iron uptake, its binding affinity for Tf is 25-fold less than that of TfR1 (Kawabata et al., 1999; West et al., 2000). This difference in affinity may enhance the role of TfR2 as an iron sensor, allowing it to be sensitive to changes in Tf saturation in the blood. The lower affinity of TfR2 does not seem to diminish its ability to endocytose iron. In TRVb cells lacking both TfR1 and TfR2, transfection of TfR2 increased Tf- mediated ^55^Fe uptake to similar levels as transfected TfR1(Kawabata et al., 1999). TfR1 is expressed in many tissues whereas *TfR2* expression is limited to the liver and erythropoietic progenitors (Sposi et al., 2000). The limited expression of *TfR2* may explain why deletion of TfR1 is embryonic lethal (Levy et al., 1999). While both TfR1 and TfR2 bind and endocytose Tf, their different affinity for Tf and different expression patterns suggest different functions.

Other differences exist which explain the inability of TfR2 to replace TfR1. TfR1 and TfR2 are differentially regulated by iron and holo-Tf. Iron response elements (IRE’s) on the 3′ TfR1 mRNA account for the rapid turnover of TfR1 mRNA under high iron conditions, which functions to reduce iron import (Owen and Kuhn, 1987). While TfR1 mRNA levels respond quickly to iron levels it is a relatively stable protein with a turnover of ~24 h. Therefore, the response of cells to high intracellular iron by downregulation of TfR1 is relatively slow. In contrast, *TfR2* lacks the IRE’s for the regulation of its mRNA by intracellular iron and at the protein level, turns over much faster. The binding of Tf to TfR2 regulates both its stability and its trafficking within cells (Johnson and Enns, 2004; Johnson et al., 2007). In the presence of holo-Tf, TfR2 levels are increased by redirection of TfR2 to the recycling endosomes, which increases its stability (Johnson and Enns, 2004; Robb and Wessling-Resnick, 2004; Chen et al., 2009). These differences are the result of very distinct cytoplasmic domains. The TfR1 and TfR2 cytoplasmic domains both have a YXXφ-based endocytic motif for clathrin-mediated endocytosis, but share little else. In addition to the YXXφ motif, TfR2 also has a phosphofurin acidic cluster sorting-1 (PACS-1) motif and coprecipitates with the PACS-1 protein (Chen et al., 2009). This motif is most likely responsible for the Tf-dependent recycling of TfR2 from endosomes to the cell surface (Chen et al., 2009). Human TfR2 is glycosylated at three sites: 240, 339, and 754. This glycosylation is necessary for the Tf-induced stabilization of TfR2, but does not affect its ability to bind Tf or its trafficking to the cell surface (Zhao and Enns, 2013). Despite their structural similarity and ability to bind Tf, the differences in Tf-induced stability and the cytoplasmic domains of TfR1 and TfR2 indicate that they both handle and are affected by Tf differently.

In addition to functional differences in Tf handling, TfR1 and TfR2 appear to interact with the original hereditary hemochromatosis protein (HFE) through alternate domains. TfR1 and HFE interact through the helical domain of TfR1 and the α1 and α2 domains of HFE (Bennett et al., 2000). Tf and HFE compete with each other for binding to TfR1 because they have overlapping binding sites (Giannetti et al., 2003; Giannetti and Bjorkman, 2004). TfR2 and HFE interact through the TfR2 stalk region between residues 104 and 250 and the HFE α3 domain (Chen et al., 2007; D’Alessio et al., 2012). The binding sites of HFE and Tf do not appear to overlap in TfR2 (Chen et al., 2007). This lends itself to the hypothesis that Tf-binding to TfR1 releases HFE, making it available to functionally interact with TfR2. Coprecipitation studies indicate that TfR2 and HFE interact readily, however, TfR2/HFE interaction remains controversial as coprecipitation of endogenous Tfr2 from liver lysates expressing myc-tagged Hfe did not yield positive results (Chen et al., 2007; Schmidt and Fleming, 2012). However, in terms of functionality, it appears that both TfR2 and HFE are needed for Tf-sensing (Gao et al., 2010). In addition to the binding of HFE and Tf, a recent report has found an interaction between TfR2 and the BMP co-receptor, HJV, which is an interesting link between the TfR2/HFE complex and BMP-signaling (D’Alessio et al., 2012). The ability of HFE, TfR2, and HJV to form a complex *in vitro*, coupled with the fact that mutations in any one of these proteins causes HH suggests a role for this complex in the regulation of hepcidin. This is consistent with the different roles of TfR1 and TfR2. TfR1 regulates cellular iron uptake and TfR2 senses iron levels and regulates body iron uptake.

## DISEASE-CAUSING MUTATIONS IN TfR2

TfR2 mutations result in the disease HH. Unlike HFE HH, which seems to have for the most part risen from a single amino acid HFE mutation and spread throughout Europe, TfR2 HH is far rarer and is the result of various mutations. The first reported TfR2 mutation, the truncation mutant *Y250X*, was found in two unrelated Sicilian families (Camaschella et al., 2000). Since then, a variety of other TfR2 mutations have been found in Italian patients (Majore et al., 2006; Biasiotto et al., 2008; Gerolami et al., 2008; Radio et al., 2014). Because the most common mutation in HFE is not present in the Japanese population, Japanese patients with HH most frequently have mutations in TfR2 (Hattori et al., 2003; Koyama et al., 2005; Hayashi et al., 2006). Many mutations in TfR2 identified to date fail to give insight into TfR2 function because most mutations result in misfolded proteins that remain in the endoplasmic reticulum (ER; Wallace et al., 2008), where they are presumably degraded by the ER quality control pathway. Two interesting point mutations, Q890P and M172K (predicted to disrupt Tf binding and HFE binding respectively; Mattman et al., 2002; Roetto et al., 2010), fail to reach the cell surface in analogous mouse mutations (Q685P and M167K; Wallace et al., 2008). TfR2 mutations continue to be found in patients around the world (Le Gac et al., 2004; Hsiao et al., 2007; Zamani et al., 2012)

## HEPCIDIN REGULATION

Hepcidin is a primary regulator of total body iron homeostasis and disease-causing TfR2 mutations cause HH through a reduction in hepcidin transcription. The transcription of hepcidin is regulated by iron, bone morphogenetic proteins (BMPs), inflammation, hypoxia, and erythropoietic activity. The hepcidin promoter has two BMP response elements (REs), the distal BMP RE2 and the proximal RE1 (Verga Falzacappa et al., 2008; Truksa et al., 2009). Within the proximal BMP RE1 lies a signal transducer and activator of transcription3 (STAT3) binding site that is responsible for upregulation of hepcidin in response to inflammation (Verga Falzacappa et al., 2008). Interestingly, response of hepcidin to inflammation requires the BMP-binding element in RE1 be intact as well as the STAT binding site, indicating that BMP signaling may be required to keep the chromatin open for STAT binding (Wang et al., 2005; Casanovas et al., 2009) Within the distal BMP RE2, lies a hepatocyte-specific hepatocyte nuclear factor 4 alpha (HNF4α) binding site as well as bZIP (basic leucine zipper domain) and COUP (chicken ovalbumin upstream promoter transcription factor) motifs indicating that hepcidin transcription relies on a set of transcription factors, including ones that are tissue specific (Truksa et al., 2009). Stimulation of hepcidin in response to BMP signaling and HJV expression requires that both BMP RE1 and BMP RE2 be intact. The distal BMP RE2 is required for hepcidin response to iron levels (Truksa et al., 2007, 2009). While hepcidin can respond to inflammation and hypoxia, it seems that regulation of hepcidin expression requires BMP-signaling.

While BMP’s 2, 4, and 6 are all expressed in the liver and are capable of stimulating hepcidin expression in hepatocytes and hepatoma cell lines, BMP-6 is the only one that is positively regulated by iron (Kautz et al., 2008), and it is expressed mainly in the endothelial cells of the liver (Knittel et al., 1997; Zhang et al., 2011; Enns et al., 2013). Deletion of BMP-6 results in severe iron-overload, confirming its role in iron homeostasis (Andriopoulos et al., 2009; Meynard et al., 2009). BMP-6 also interacts with the BMP co-receptor HJV (Andriopoulos et al., 2009). This indicates that while other BMP’s may have an effect on basal hepcidin expression, it is BMP-6 that is regulated by iron, and functions as a regulator of iron homeostasis. As of yet, how BMP-6 is regulated by iron is not known. Deletion of BMP-6 has little effect on bone formation, with only a slight delay in sternal ossification (Solloway et al., 1998), indicating that BMP-6 may function primarily as a regulator of iron homeostasis.

BMP-signaling requires binding of BMP’s to BMP-receptors. BMP-receptors are arranged as a tetramer of two type I and type II receptors. The type II receptors phosphorylate the type I receptors upon ligand binding, and the phosphorylated receptor can then phosphorylate and activate intracellular receptor-associated SMADs (R-SMADs), which then bind the co-SMAD, SMAD4 to then enter the nucleus and regulate transcription (Wrana et al., 1992; Lagna et al., 1996; Macias-Silva et al., 1996; Souchelnytskyi et al., 1996). In the liver, HJV binds to the BMP type II receptor ActRIIa to enhance BMP-signaling and hepcidin expression (Xia et al., 2008). Two BMP type I receptors are involved in hepcidin regulation, Alk2 and Alk3 (Babitt et al., 2006; Xia et al., 2008; Steinbicker et al., 2011). The Alk3 receptor is necessary for basal hepcidin expression in mice and deletion of Alk3 has a more severe iron-overload phenotype than Alk2, however, Alk2 seems to be necessary for the response of hepcidin to iron and HJV (Steinbicker et al., 2011). Therefore, the ligand BMP-6, the BMP co-receptor HJV, and BMP receptors ActRIIa, Alk2, and Alk3 all make up the liver BMP-signaling pathway.

The BMP-signaling pathway can also be modulated by the inhibitory SMADs (iSMADs), SMAD6 and SMAD7. These iSMADs are part of a negative feedback loop and are induced by BMP signaling. They inhibit BMP-signaling by binding to BMP receptors (which inhibits SMAD phosphorylation), recruiting ubiquitin ligases (to induce degradation of the receptors), or they can enter the nucleus and disrupt binding of phosphorylated SMADs to target genes (Hayashi et al., 1997; Kavsak et al., 2000; Zhang et al., 2007). In keeping with their role as negative-feedback loop inhibitors of BMP-signaling, SMAD6 and SMAD7 are co-regulated with hepcidin and SMAD7 is upregulated in response to iron (Kautz et al., 2008; Vujic Spasic et al., 2013). In addition, SMAD7 can modulate signaling by directly binding the promoter region and inhibiting hepcidin expression (Mleczko-Sanecka et al., 2010).

Deletion of HFE, HJV, or TfR2 results in a reduction of phosphorylated SMADs 1/5/8, indicating that reduced hepcidin expression is mediated through the BMP-signaling pathway (Babitt et al., 2006; Corradini et al., 2009, 2011). Presumably, HJV regulates BMP-signaling through enhancing binding of BMP ligand to BMP-receptors and promoting the assembly of the BMP-signaling complex (Babitt et al., 2006). The severe phenotype of HJV knockout mice and juvenile hemochromatosis patients is in keeping with the important role of HJV as a BMP co-receptor and with the importance of the BMP-signaling pathway in basal hepcidin transcription. HFE and TfR2 mutations are far less severe, indicating that they are involved in fine-tuning of iron levels. How HFE and TfR2 modulate hepcidin expression through the BMP-signaling pathway is not understood.

## PHYSIOLOGICAL FUNCTION OF TfR2

TfR2 has been hypothesized to be the Tf-sensor since the discovery of its disease-causing mutations (Camaschella et al., 2000; Fleming et al., 2000; Roetto et al., 2001). Wild type mouse primary hepatocytes, when treated with holo-Tf, will respond within 24 h by a 2-fold upregulation of hepcidin expression and wild type mice injected with iron will also see this increase in hepcidin levels (Kawabata et al., 2005; Lin et al., 2007; Ramey et al., 2009; Ramos et al., 2011). This mirrors the ~2-fold increase in urinary hepcidin seen in humans who were challenged with iron and had a corresponding increase in Tf-saturation (Lin et al., 2007; Girelli et al., 2011). In contrast, Tfr2 mutant mouse primary hepatocytes do not respond to treatment, indicating a role of Tfr2 in Tf-sensitivity (Gao et al., 2009). In addition, deletion of the Tfr2 binding partner, Hfe, also results in loss of Tf- sensitivity, indicating that it may be the TfR2/HFE complex that is involved in iron-sensing (Gao et al., 2009). In human patients with TfR2 HH, urinary hepcidin levels do not respond to iron challenge, and HFE HH patients have a blunted hepcidin response, indicating that both molecules are needed in order to modulate iron uptake in response to dietary iron (Girelli et al., 2011). The 2-fold hepcidin response to holo-Tf in primary hepatocytes is physiological, as HFE HH patients only have a 2-fold difference in hepcidin levels and the disease results in the slow accumulation of iron over the lifetime of the individual (Van Dijk et al., 2008).

While mutations in TfR2 and HFE both result in a slow disease progression, loss of TfR2 appears to be more severe than loss of HFE. There is a reported case of juvenile hemochromatosis resulting from TfR2 mutation and serum hepcidin levels are lower in TfR2 HH patients (Nemeth et al., 2005; Pietrangelo et al., 2005; Girelli et al., 2011). Because of the scarcity of TfR2 HH patients in contrast to HFE HH patients, it is hard to compare severity of HFE and TfR2 mutations. Tfr2 mutant mice of the same genetic background as Hfe^-/-^ mice have higher iron accumulation than Hfe^-/-^ mice (Wallace et al., 2009). Mice and humans lacking both Tfr2 and Hfe have a more severe phenotype than either single mutation (Pietrangelo et al., 2005; Wallace et al., 2009; Corradini et al., 2011), indicating that either one or both proteins may have alternate functions, or that the complex may be able to partially function with one member missing. Transfection of HFE into cell lines that do and do not express TfR2 decreases iron uptake indicating that HFE almost certainly has another function than that of the TfR2/HFE complex (Roy et al., 1999; Wang et al., 2003; Carlson et al., 2005). Other responses that can be attributed to TfR2 remain unknown.

The existence and role of the Tfr2/Hfe complex is not without controversy. While Tfr2 and Hfe immunoprecipitate readily in transfected cells, one report was unable to confirm interaction with endogenous Tfr2 in primary hepatocytes expressing myc-tagged Hfe transgene (Schmidt and Fleming, 2012). In addition, reports differ as to whether Hfe overexpression in Tfr2 mutant mice can increase hepcidin levels and reduce iron accumulation in mice (Gao et al., 2010; Schmidt and Fleming, 2012). These results are further complicated by the ability of Hfe to affect iron uptake, as chronic higher iron stores lead to increased BMP-6 expression independently of either HFE or TfR2. BMP-6 expression is not however, dependent on Tf-saturation. Tf-deficient mice have high iron stores, despite being anemic (Trenor et al., 2000). In keeping with their high tissue iron levels, these mice have increased BMP-6 levels while hepcidin levels are still below normal (Bartnikas et al., 2011; Bartnikas and Fleming, 2012), indicating that Tf is a necessary part of the BMP-signaling pathway that leads to hepcidin expression. Tf-deficient mice that are treated with Tf increase hepcidin expression, however, this increase in hepcidin expression is attenuated when Hjv is also deleted (Bartnikas and Fleming, 2012), indicating that the hepcidin response to Tf requires HJV. Experiments in isolated primary hepatocytes get around the complication of BMP-6 expression, because BMP-6 is expressed in the endothelial cells. In primary hepatocytes, both Hfe and Tfr2 are needed for a hepcidin response to holo-Tf, indicating that, at least in regards to blood iron-sensing, both proteins are required.

While the mechanism by which TfR2 affects hepcidin expression through the BMP-signaling pathway remains nebulous, its ability to bind Tf and its requirement for the hepcidin response to holo-Tf makes it likely that TfR2 is the Tf- sensor. That it also interacts with HFE, and that HFE is also required for the response of hepcidin to holo-Tf provides a strong indication that TfR2 senses iron as part of a TfR2/HFE complex, and that this complex formation is important to body iron homeostasis. The further binding of both HFE and TfR2 to the BMP co-receptor, HJV, provides a possible link between the TfR2/HFE Tf-sensing complex and the BMP-signaling complex, and further research is needed to ascertain the functionality of this complex.

## CURRENT TfR2 MODELS

**FIGURE 1:**
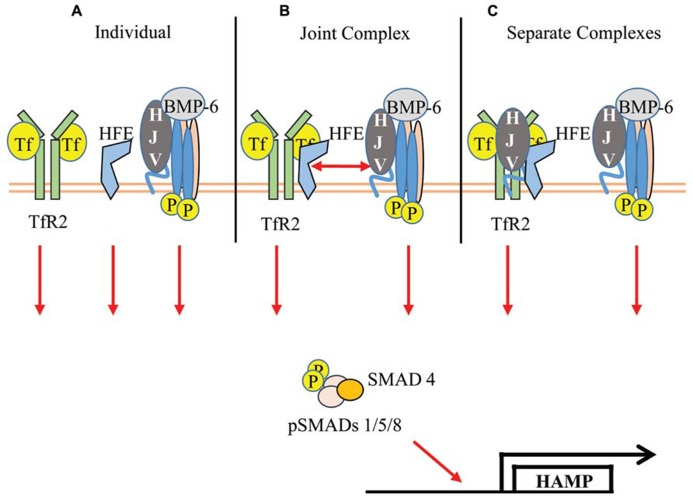
**Models of Tf/TfR2-induced upregulation of hepcidin transcription.** **(A)** TfR2 and HFE act independently to increase pSMAD induction of hepcidin transcription. **(B)** Tf induces the formation of a large complex between TfR2/HFE/HJV/BMPR’s to enhance pSMAD. **(C)** Separate complexes composed of Tf/TfR2/HFE/HJV and HJV/BMP-6/BMPR’s signal to enhance pSMAD.

The controversy regarding the TfR2/HFE complex, coupled with the new report of a TfR2/HFE/HJV complex lends itself to three possible models for the Tf-sensitive regulation of hepcidin by TfR2 (**Figure 1**). First, if TfR2 and HFE do not functionally interact, then Tf/TfR2, HFE, and the BMP-signaling complex affect pSMAD levels independently of one another. Support for this model lies in the increased severity of the Tfr2-Hfe double knockout mouse (Wallace et al., 2009) and the failed interaction of the myc-tagged Hfe transgene with endogenous Tfr2 (Schmidt and Fleming, 2012). Second, if reports of TfR2/HFE and TfR2/HFE/HJV interactions are functionally significant, then the TfR2/HFE complex could interact with the BMP-signaling complex upon Tf-binding, thereby affecting pSMAD levels. Support for this model lies in the reports of TfR2/HFE interaction (Goswami and Andrews, 2006; Chen et al., 2007; D’Alessio et al., 2012), the requirement of both Tfr2 and Hfe for Tf-sensitivity (Gao et al., 2009, 2010), and the recent report of a TfR2/HFE/HJV complex (D’Alessio et al., 2012). The third model proposes that HJV interacts with both the TfR2/HFE complex and the BMP-signaling complex, and both of these complexes affect pSMAD levels independently. While the functional significance of the TfR2/HFE complex or the TfR2/HFE/HJV complex may still be up for debate, TfR2 plays an important role in regulating hepcidin levels in response to holo-Tf through the BMP-signaling pathway.

## SUMMARY

TfR2 plays an important role in the fine-tuning of body iron uptake and loss of TfR2 function leads to Type III HH. TfR2 senses changes in blood-iron levels through its interaction with holo-Tf. While it is similar in structure to the iron-endocytosis protein, TfR1, it has a lower affinity for Tf, an alternate binding site for HFE, and is differentially trafficked and regulated. These differences, along with the tissue expression pattern of TfR2, indicate that the function of TfR1 is to bind and endocytose iron for cellular purposes, while the function of TfR2 is to sense blood iron levels. TfR2 is able to regulate body iron uptake in response to blood iron levels by modulating hepcidin expression through the BMP-signaling pathway. The formation and functional significance of the TfR2/HFE complex remains controversial, but both proteins are necessary for Tf-sensitivity. The interaction of TfR2/HFE with the BMP co-receptor, HJV, may provide an interesting link between TfR2, HFE, and the BMP-signaling pathway.

## Conflict of Interest Statement

The authors declare that the research was conducted in the absence of any commercial or financial relationships that could be construed as a potential conflict of interest.
